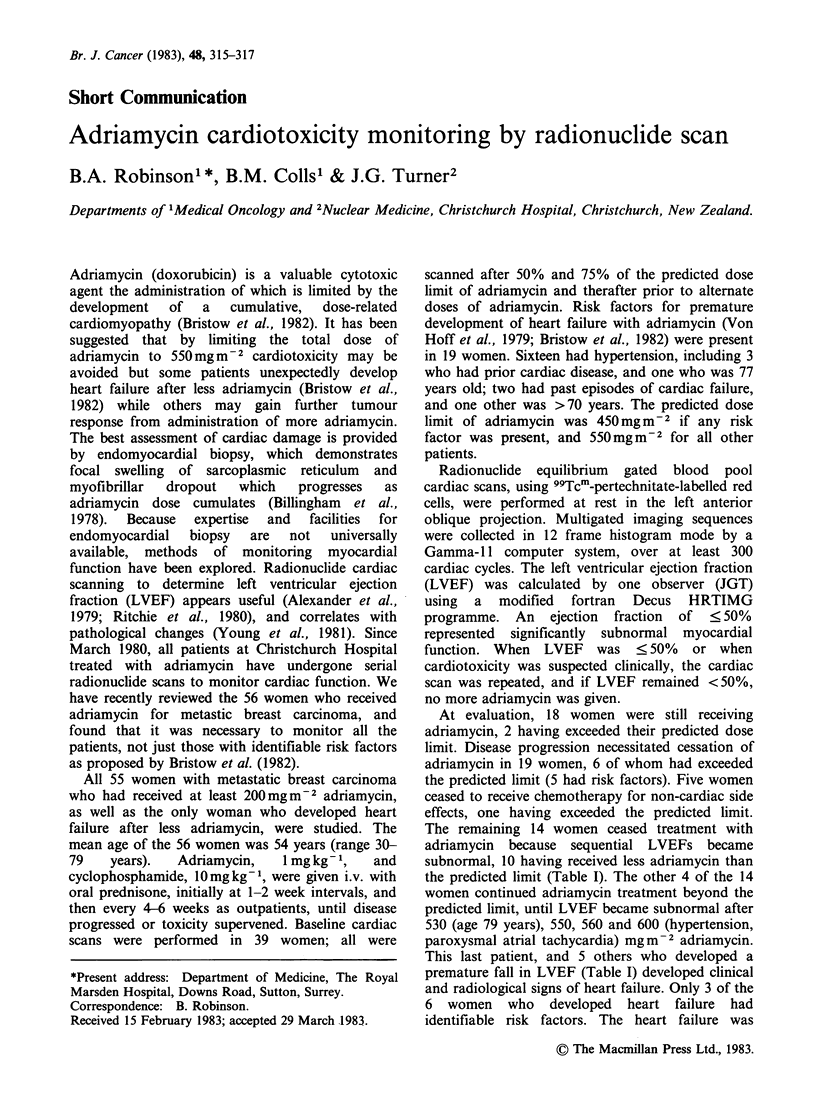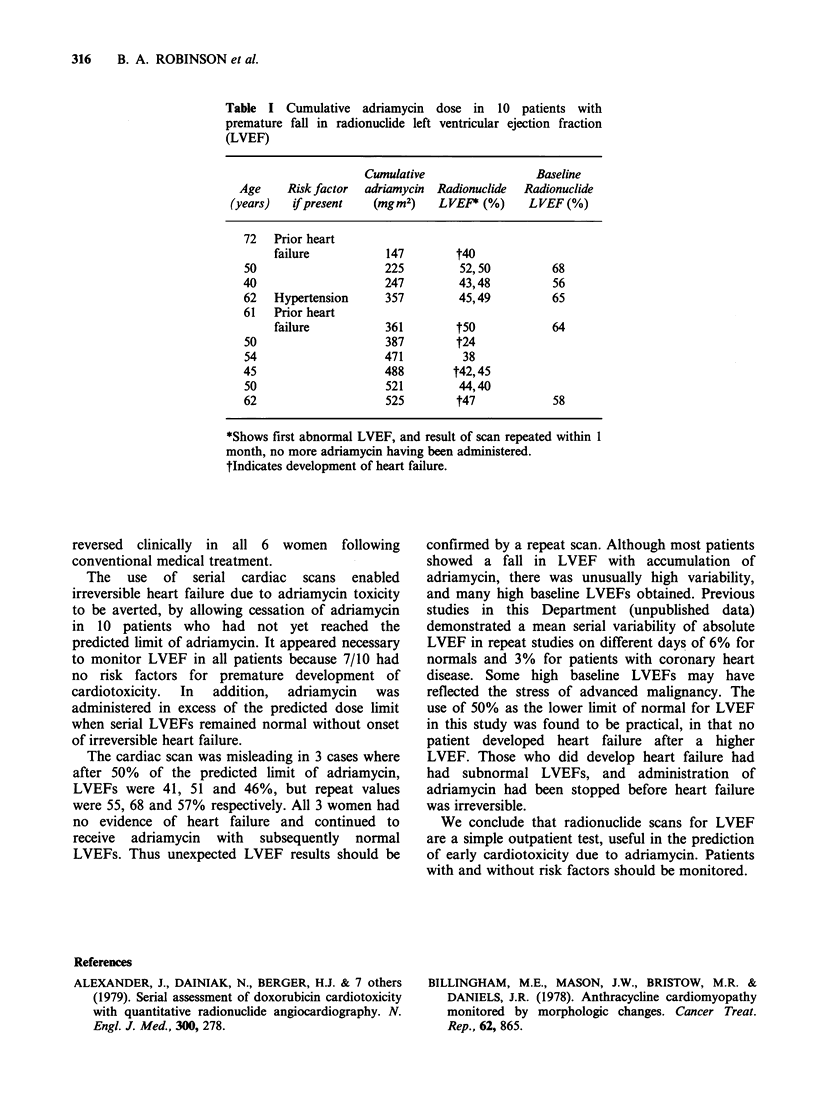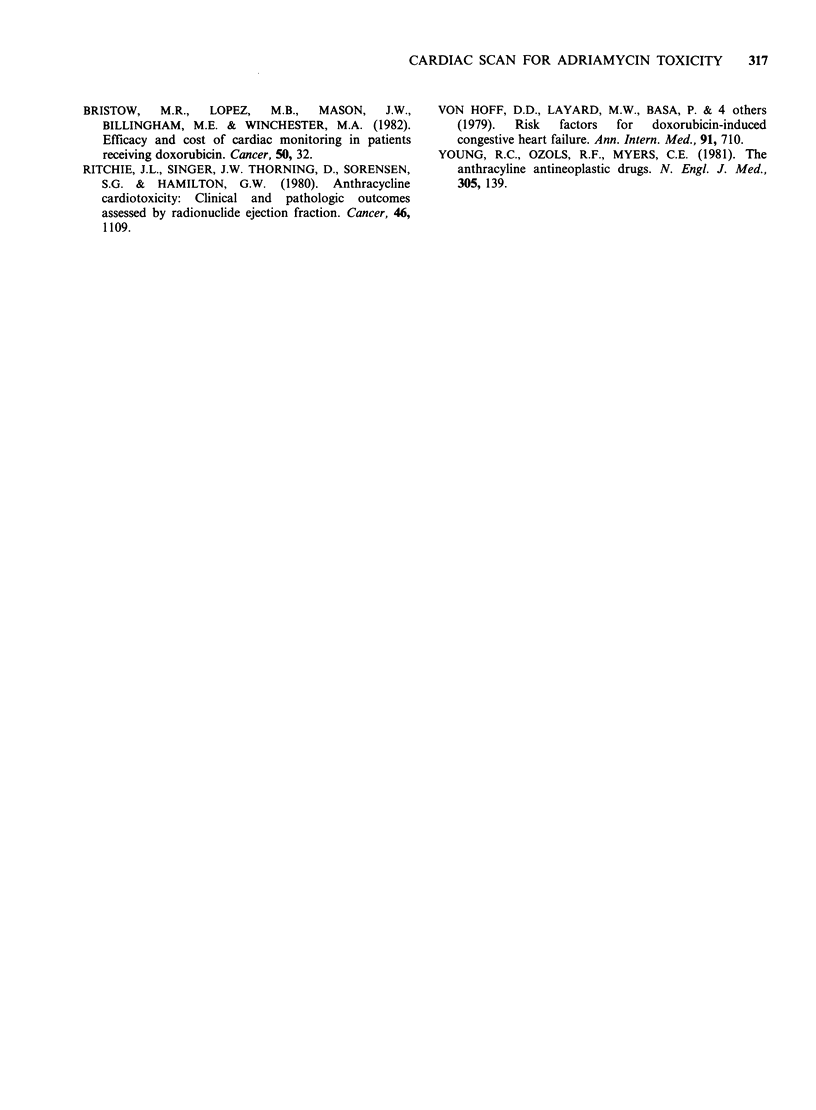# Adriamycin cardiotoxicity monitoring by radionuclide scan.

**DOI:** 10.1038/bjc.1983.188

**Published:** 1983-08

**Authors:** B. A. Robinson, B. M. Colls, J. G. Turner


*Br. J. Cancer* (1983), **48**, 315–317

### Short Communication

### Adriamycin cardiotoxicity monitoring by radionuclide scan

B.A. Robinson[1]*, B.M. Colls[1] & J.G. Turner[2]

*Departments of [1]Medical Oncology and [2]Nuclear Medicine, Christchurch Hospital, Christchurch, New Zealand.*

Adriamycin (doxorubicin) is a valuable cytotoxic agent the administration of which is limited by the development of a cumulative, dose-related cardiomyopathy (Bristow *et al.*, 1982). It has been suggested that by limiting the total dose of adriamycin to $550\,mg\,m^{-2}$ cardiotoxicity may be avoided but some patients unexpectedly develop heart failure after less adriamycin (Bristow *et al.*, 1982) while others may gain further tumour response from administration of more adriamycin. The best assessment of cardiac damage is provided by endomyocardial biopsy, which demonstrates focal swelling of sarcoplasmic reticulum and myofibrillar dropout which progresses as adriamycin dose cumulates (Billingham *et al.*, 1978). Because expertise and facilities for endomyocardial biopsy are not universally available, methods of monitoring myocardial function have been explored. Radionuclide cardiac scanning to determine left ventricular ejection fraction (LVEF) appears useful (Alexander *et al.*, 1979; Ritchie *et al.*, 1980), and correlates with pathological changes (Young *et al.*, 1981). Since March 1980, all patients at Christchurch Hospital treated with adriamycin have undergone serial radionuclide scans to monitor cardiac function. We have recently reviewed the 56 women who received adriamycin for metastic breast carcinoma, and found that it was necessary to monitor all the patients, not just those with identifiable risk factors as proposed by Bristow *et al.* (1982).

All 55 women with metastatic breast carcinoma who had received at least $200\,mg\,m^{-2}$ adriamycin, as well as the only woman who developed heart failure after less adriamycin, were studied. The mean age of the 56 women was 54 years (range 30–79 years). Adriamycin, $1\,mg\,kg^{-1}$, and cyclophosphamide, $10\,mg\,kg^{-1}$, were given i.v. with oral prednisone, initially at 1–2 week intervals, and then every 4–6 weeks as outpatients, until disease progressed or toxicity supervened. Baseline cardiac scans were performed in 39 women; all were scanned after 50% and 75% of the predicted dose limit of adriamycin and therafter prior to alternate doses of adriamycin. Risk factors for premature development of heart failure with adriamycin (Von Hoff *et al.*, 1979; Bristow *et al.*, 1982) were present in 19 women. Sixteen had hypertension, including 3 who had prior cardiac disease, and one who was 77 years old; two had past episodes of cardiac failure, and one other was >70 years. The predicted dose limit of adriamycin was $450\,mg\,m^{-2}$ if any risk factor was present, and $550\,mg\,m^{-2}$ for all other patients.

Radionuclide equilibrium gated blood pool cardiac scans, using $^{99}Tc^{m}$-pertechnitate-labelled red cells, were performed at rest in the left anterior oblique projection. Multigated imaging sequences were collected in 12 frame histogram mode by a Gamma-11 computer system, over at least 300 cardiac cycles. The left ventricular ejection fraction (LVEF) was calculated by one observer (JGT) using a modified fortran Decus HRTIMG programme. An ejection fraction of $\leq 50\%$ represented significantly subnormal myocardial function. When LVEF was $\leq 50\%$ or when cardiotoxicity was suspected clinically, the cardiac scan was repeated, and if LVEF remained $<50\%$, no more adriamycin was given.

At evaluation, 18 women were still receiving adriamycin, 2 having exceeded their predicted dose limit. Disease progression necessitated cessation of adriamycin in 19 women, 6 of whom had exceeded the predicted limit (5 had risk factors). Five women ceased to receive chemotherapy for non-cardiac side effects, one having exceeded the predicted limit. The remaining 14 women ceased treatment with adriamycin because sequential LVEFs became subnormal, 10 having received less adriamycin than the predicted limit (Table I). The other 4 of the 14 women continued adriamycin treatment beyond the predicted limit, until LVEF became subnormal after 530 (age 79 years), 550, 560 and 600 (hypertension, paroxysmal atrial tachycardia) $mg\,m^{-2}$ adriamycin. This last patient, and 5 others who developed a premature fall in LVEF (Table I) developed clinical and radiological signs of heart failure. Only 3 of the 6 women who developed heart failure had identifiable risk factors. The heart failure was

*Present address: Department of Medicine, The Royal Marsden Hospital, Downs Road, Sutton, Surrey.
Correspondence: B. Robinson.
Received 15 February 1983; accepted 29 March 1983.

© The Macmillan Press Ltd., 1983.

**Table I** Cumulative adriamycin dose in 10 patients with premature fall in radionuclide left ventricular ejection fraction (LVEF)

| *Age (years)* | *Risk factor if present* | *Cumulative adriamycin ($mg\,m^2$)* | *Radionuclide LVEF** (%) | *Baseline Radionuclide LVEF* (%) |
|---|---|---|---|---|
| 72 | Prior heart failure | 147 | †40 | |
| 50 | | 225 | 52, 50 | 68 |
| 40 | | 247 | 43, 48 | 56 |
| 62 | Hypertension | 357 | 45, 49 | 65 |
| 61 | Prior heart failure | 361 | †50 | 64 |
| 50 | | 387 | †24 | |
| 54 | | 471 | 38 | |
| 45 | | 488 | †42, 45 | |
| 50 | | 521 | 44, 40 | |
| 62 | | 525 | †47 | 58 |

*Shows first abnormal LVEF, and result of scan repeated within 1 month, no more adriamycin having been administered.
†Indicates development of heart failure.

reversed clinically in all 6 women following conventional medical treatment.

The use of serial cardiac scans enabled irreversible heart failure due to adriamycin toxicity to be averted, by allowing cessation of adriamycin in 10 patients who had not yet reached the predicted limit of adriamycin. It appeared necessary to monitor LVEF in all patients because 7/10 had no risk factors for premature development of cardiotoxicity. In addition, adriamycin was administered in excess of the predicted dose limit when serial LVEFs remained normal without onset of irreversible heart failure.

The cardiac scan was misleading in 3 cases where after 50% of the predicted limit of adriamycin, LVEFs were 41, 51 and 46%, but repeat values were 55, 68 and 57% respectively. All 3 women had no evidence of heart failure and continued to receive adriamycin with subsequently normal LVEFs. Thus unexpected LVEF results should be confirmed by a repeat scan. Although most patients showed a fall in LVEF with accumulation of adriamycin, there was unusually high variability, and many high baseline LVEFs obtained. Previous studies in this Department (unpublished data) demonstrated a mean serial variability of absolute LVEF in repeat studies on different days of 6% for normals and 3% for patients with coronary heart disease. Some high baseline LVEFs may have reflected the stress of advanced malignancy. The use of 50% as the lower limit of normal for LVEF in this study was found to be practical, in that no patient developed heart failure after a higher LVEF. Those who did develop heart failure had had subnormal LVEFs, and administration of adriamycin had been stopped before heart failure was irreversible.

We conclude that radionuclide scans for LVEF are a simple outpatient test, useful in the prediction of early cardiotoxicity due to adriamycin. Patients with and without risk factors should be monitored.

### References

ALEXANDER, J., DAINIAK, N., BERGER, H.J. & 7 others (1979). Serial assessment of doxorubicin cardiotoxicity with quantitative radionuclide angiocardiography. *N. Engl. J. Med.*, **300**, 278.

BILLINGHAM, M.E., MASON, J.W., BRISTOW, M.R. & DANIELS, J.R. (1978). Anthracycline cardiomyopathy monitored by morphologic changes. *Cancer Treat. Rep.*, **62**, 865.

BRISTOW, M.R., LOPEZ, M.B., MASON, J.W., BILLINGHAM, M.E. & WINCHESTER, M.A. (1982). Efficacy and cost of cardiac monitoring in patients receiving doxorubicin. *Cancer,* **50,** 32.

RITCHIE, J.L., SINGER, J.W. THORNING, D., SORENSEN, S.G. & HAMILTON, G.W. (1980). Anthracycline cardiotoxicity: Clinical and pathologic outcomes assessed by radionuclide ejection fraction. *Cancer,* **46,** 1109.

VON HOFF, D.D., LAYARD, M.W., BASA, P. & 4 others (1979). Risk factors for doxorubicin-induced congestive heart failure. *Ann. Intern. Med.,* **91,** 710.

YOUNG, R.C., OZOLS, R.F., MYERS, C.E. (1981). The anthracyline antineoplastic drugs. *N. Engl. J. Med.,* **305,** 139.